# Effective treatment of peritoneal myeloid sarcoma with decitabine and venetoclax: A case report

**DOI:** 10.1016/j.lrr.2025.100534

**Published:** 2025-08-08

**Authors:** Kavya Sudireddy, Minorvi Amin, Rafy Odeh, Patrick Svrcek, Salwa Khedr, Lloyd Hutchinson, Shyam A. Patel, Jan Cerny, Laurie Pearson

**Affiliations:** a Department of Medicine, UMass Chan Medical School, 55 Lake Avenue North, Worcester, MA 01655, USA; b Department of Neurology, UMass Chan Medical School, 55 Lake Avenue North, Worcester, MA 01655, USA; c Department of Pathology, UMass Chan Medical School, 55 Lake Avenue North, Worcester, MA 01655, USA; d Department of Radiology, UMass Chan Medical School, 55 Lake Avenue North, Worcester, MA 01655, USA; e Division of Hematology/Oncology, Department of Medicine, UMass Chan Medical School, 55 Lake Avenue North, Worcester, MA 01655, USA

**Keywords:** Peritoneal myeloid sarcoma, Extramedullary AML, Hypomethylating agents, Venetoclax

## Abstract

Myeloid sarcomas (MS) are rare extramedullary manifestations of myeloid progenitor cells occurring with or without acute myeloid leukemia. Peritoneal MS is exceptionally uncommon, with no established treatment guidelines, but treatment has historically consisted of cytotoxic chemotherapy. We present the case of a 78-year-old female with 60% CD34+, HLA-DR+, and CD33+ myeloblasts in ascitic fluid, while bone marrow biopsy demonstrated only molecular evidence of the leukemic clone with *FLT3-ITD, ASXL1*, and *TET2* mutations. Findings were consistent with primary peritoneal myeloid sarcoma. Due to chemotherapy ineligibility, the patient was treated with decitabine and venetoclax. After nine cycles, she demonstrated a complete radiographic response. To our knowledge, this is the first case report of a patient with primary peritoneal MS treated with a combination of a hypomethylating agent and venetoclax.

## 1 Intro/Background

Myeloid sarcomas (MS) are rare presentations of myeloid progenitor cell masses in extramedullary sites, most common in the lymph nodes, bone, skin (leukemia cutis), digestive tract, breast, and nervous system [[1], [2], [3]].

Peritoneal MS is extremely rare, with no established guidelines for therapy in elderly patients (>65 years of age) [[4], [5], [6], [7]]. Although conventional AML regimens have been used, therapeutic efficacy and tolerability are variable [2,6].

MS exhibits diverse molecular features; abdominal MS has been associated with CBFB-MYH11 chromosomal fusion, the *FLT3-ITD* mutation, and/or RTK-RAS pathway mutations [8,9].

Here, we report a case of a patient with primary peritoneal MS, with only molecular evidence of the leukemic clone in the marrow, who was treated with a hypomethylating agent (HMA) and venetoclax.

## 2 Clinical presentation

**Fig. 1:**
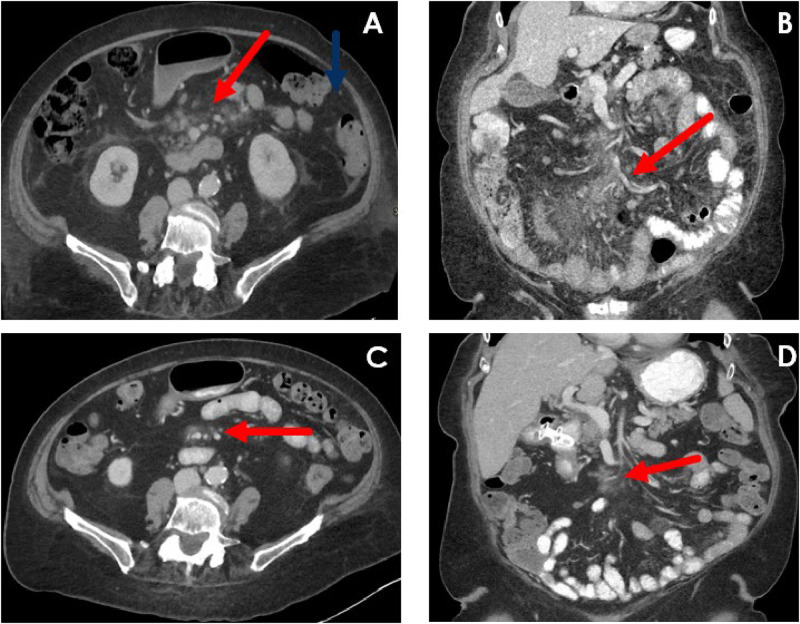
(A) Contrast-enhanced axial CT of the abdomen and pelvis demonstrates increased central mesenteric attenuation (“misty mesentery”), mesenteric lymphadenopathy (red arrow), as well as mesenteric soft tissue edema/infiltrate (blue arrow). The central mesentery involvement can be best appreciated on the coronal reformat (B, red arrow). After initiation of therapy, there is substantial decrease in the degree of mesenteric involvement with notable decrease in size and conspicuity of mesenteric lymph nodes (C, red arrow). The overall extent of mesenteric improvement is best appreciated on the coronal plane (D, red arrow). Findings are consistent with treatment effect.

A 78-year-old female with a history of emphysema, hypertension, and osteoporosis presented to an outside hospital with 6 months of abdominal pain progressing to vomiting, obstipation, and diarrhea. CT of the abdomen and pelvis revealed multiple areas of asymmetric wall thickening in the jejunum and ileum, mesenteric edema, and adenopathy. The patient was discharged with a presumptive diagnosis of gastroenteritis. One month later, symptoms recurred, and repeat CT abdomen and pelvis showed interval progression of bowel thickening and adenopathy with new splenomegaly and ascites (Fig. 1a).

**Fig. 2:**
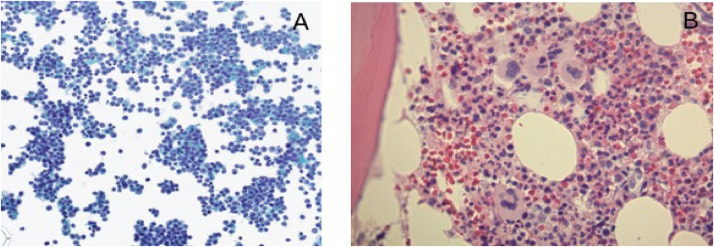
(A) Cytology smear of the peritoneal fluid showing numerous immature discohesive cells with high nucleocytoplasmic ratio, immature nuclear chromatin, and prominent nucleoli (Pap stain, x200). (B) Bone marrow biopsy showing increased megakaryocytes with atypical and hyper-segmented nuclei in occasional clusters (Hematoxylin and Eosin, x400).

Diagnostic paracentesis revealed abundant, discohesive CD34+ blast cells with a high nucleocytoplasmic ratio, open chromatin, and prominent nucleoli (Fig. 2a). Ascitic fluid flow cytometry revealed 60 % myeloblasts positive for CD34, HLA-DR, and CD33. Bone marrow was hypercellular with increased and atypical megakaryocytes and occasional erythroid and myeloid dysplasia in <10 % of cells (Fig. 2b). Myeloblasts were not increased at 1 %. Next Generation Sequencing (NGS) Panel was positive for *FLT3-ITD* (VAF 0–1 %), *ASXL1* (VAF 1–5 %), and *TET2* mutations (VAF 1–5 %), but negative for *NPM1*. Chromosomal analysis revealed a normal karyotype (46, XX). Overall, these findings were consistent with primary peritoneal MS with minimal medullary involvement.

Given advanced age and comorbidities, the patient was treated with decitabine 20mg/m^2 on days 1–5 and venetoclax. Duration of venetoclax was abbreviated to 7 days with cycle 1 due to febrile neutropenia secondary to acute cholecystitis and *Enterococcus faecium* UTI. CT imaging at the end of cycle 1 demonstrated interval reduction in mesenteric edema and resolution of mesenteric adenopathy. Venetoclax duration was extended to 14 days with subsequent cycles. Repeat CT abdomen and pelvis at end of cycle 3 demonstrated ongoing mesenteric stranding with areas of small nodularity but no appreciable progression (Fig. 1c-d). PET/CT after cycle 5 revealed no evidence of FDG-avid metabolic disease; however, minimal stranding in the right lower quadrant was noted, suggestive of either low-volume or treated disease. Repeat bone marrow biopsy was not performed due to minimal medullary involvement at diagnosis and excellent hematologic tolerance of therapy.

Cycle frequency was decreased to every 5 weeks with cycle 5, but at the end of cycle 7 she was admitted for diarrhea with *Clostridium difficile* toxin and antigen positivity. CT imaging demonstrating colitis and reactive pelvic fluid that incompletely resolved with fidaxomicin treatment. Repeat paracentesis demonstrated atypical cells with monocytic differentiation, positive for CD33. No blasts were identified. Molecular analyses on the ascitic fluid detected mutant ASXL1 and TET2, but the *FLT3-ITD* mutation was no longer detectable, Decitabine and venetoclax were resumed every 4 weeks, and subsequent CT imaging after cycle 9 demonstrated complete resolution of bowel wall thickening and ascites.

## 3 Discussion

### 3.1 Diagnosis

MS is a rare, heterogeneous extramedullary soft tissue neoplasm composed of myeloblasts or granulocytes, with varied differentiation [4]. It can often be unifocal or multifocal and may occur as a primary extramedullary disease or as a secondary manifestation of underlying or relapsed leukemia. Diagnosing *de novo* MS in patients without bone marrow involvement or a history of leukemia is challenging. Given its tendency to be misdiagnosed as lymphoma, metastatic solid tumor, Ewing sarcoma, or other diseases, treatment may be delayed. This highlights the need for a broad differential and early collaboration with a hematopathologist to ensure accurate and timely diagnosis [3].

### 3.2 Prognosis

The prognosis for MS varies significantly. Published studies indicate that treatment approaches impact outcomes, with systemic chemotherapy showing a positive prognosis compared to local therapy, as it leads to statistically significant improvements in survival rates, although selection bias may limit comparison [2]. Additionally, increasing age is a poor prognostic factor. The median overall survival for MS patients is approximately 9 months, with a 3-year overall survival rate of 31.3 % [3].

### 3.3 Treatment

Management of MS varies based on factors such as age, comorbidities, ECOG status, and whether the sarcoma is a relapse after acute myeloid leukemia (AML) or an initial diagnosis. Treatment typically involves induction therapy with systemic chemotherapy, usually including cytarabine and anthracycline, followed by consolidation chemotherapy similar to AML regimens. Zhao et al. reported recurrence-free survival in 5 of 11 patients who underwent allogeneic hematopoietic stem cell transplantation [2].

Treatment of primary peritoneal MS has historically consisted of cytotoxic chemotherapy. Kuhlman *et al*. achieved sustained remission in a patient with primary *de novo* peritoneal MS with CBF rearrangement using 7 + 3 induction therapy followed by gemtuzumab ozogamicin and high-dose cytarabine [6]. Wang *et al*. reported complete remission in a 25-year-old man with widespread peritoneal, mesenteric, and omental infiltration using conventional AML induction with cytarabine and daunorubicin [4]. However, two other case reports involving patients with peritoneal MS who received standard chemotherapy succumbed to infectious complications [4,10].

Older patients (>age 60) often have medical contraindications to intensive chemotherapy, increased toxicity with chemotherapy, and higher risk molecular features leading to poorer response to induction chemotherapy [11]. Hypomethylating agents, such as azacitidine, decitabine, or low-dose cytarabine, have been used for AML treatment in older individuals who cannot tolerate standard induction therapy, until the paradigm shifting VIALE-A trial, in which azacitidine plus venetoclax improved overall survival compared to azacitidine alone in chemotherapy-ineligible patients with AML [12]. While this combination therapy has demonstrated promising efficacy in AML, its role in extramedullary AML remains unknown. A single case report demonstrated effective disease control and symptom resolution in a patient with bladder MS [13].

To our knowledge, this is the first case report of a patient with peritoneal MS treated with decitabine and venetoclax. Our patient’s clinical course suggests a significant disease response following the initiation of decitabine plus venetoclax.

## 4 Conclusion

In conclusion, this case report demonstrates the efficacy of HMA combined with venetoclax in managing peritoneal MS in the chemotherapy-ineligible, older population. While more data is needed to determine the standard of care in peritoneal myeloid sarcoma, this regimen may be a viable treatment option in this population.

## CRediT authorship contribution statement

**Kavya Sudireddy:** Writing – review & editing, Writing – original draft, Conceptualization. **Minorvi Amin:** Writing – review & editing, Writing – original draft, Conceptualization. **Rafy Odeh:** Data curation. **Patrick Svrcek:** Writing – review & editing, Data curation. **Salwa Khedr:** Data curation. **Lloyd Hutchinson:** Data curation. **Shyam A. Patel:** Writing – review & editing, Supervision, Conceptualization. **Jan Cerny:** Writing – review & editing. **Laurie Pearson:** Writing – review & editing, Writing – original draft, Supervision, Data curation, Conceptualization.

## Declaration of competing interest

The authors declare that they have no known competing financial interests or personal relationships that could have appeared to influence the work reported in this paper.
